# Molecular Tumor Board Improves Outcomes for Hispanic Patients With Advanced Solid Tumors

**DOI:** 10.1200/GO.23.00011

**Published:** 2024-01-18

**Authors:** Carolina Sotelo-Rodríguez, Dora Vallejo-Ardila, Alejandro Ruiz-Patiño, Diego F. Chamorro, July Rodríguez, Darwin A. Moreno-Pérez, Hernán Carranza, Jorge Otero, Carlos Vargas, Pilar Archila, Leonardo Rojas, Jairo Zuluaga, Cladelis Rubio, Camila Ordóñez-Reyes, Juan Esteban Garcia-Robledo, Sergio Mejía, Elvira Jaller, Oscar Arrieta, Andrés F. Cardona

**Affiliations:** ^1^ Foundation for Clinical and Applied Cancer Research—FICMAC, Bogotá, Colombia; ^2^ Molecular Oncology and Biology Systems Research Group (Fox-G), Universidad El Bosque, Bogotá, Colombia; ^3^ Thoracic Oncology Unit, Luis Carlos Sarmiento Angulo Cancer Treatment and Research Center (CTIC), Bogotá, Colombia; ^4^ Hematology/Oncology Division, Mayo Clinic, Scottsdale, AZ; ^5^ Thoracic Oncology Unit, Clínica Las Américas, Medellín, Colombia; ^6^ Thoracic Oncology Unit, National Cancer Institute (INCan), México City, México; ^7^ Direction of Research, Science and Education, Luis Carlos Sarmiento Angulo Cancer Treatment and Research Center (CTIC), Bogotá, Colombia

## Abstract

**PURPOSE:**

Multidisciplinary molecular tumor boards (MTBs) decode complex genomic data into clinical recommendations. Although MTBs are well-established in the oncology practice in developed countries, this strategy needs to be better explored in developing countries. Herein, we describe the possible benefits and limitations of the first MTB established in Colombia.

**METHODS:**

Demographic, clinical, and genomic information was collected between August 2020 and November 2021. By mid-2020, an MTB strategy was created to discuss clinical cases with one or more genomic alterations identified by next-generation sequencing using an open-access virtual platform. We characterized the patient population as benefiting from the recommended treatment option. We assessed the benefits and access to available targeted therapies that have the potential to change clinical management by making recommendations to treating oncologists on the basis of genomic profiling. However, we did not assess the treatment oncologists' compliance with MTB recommendations because they were not intended to replace clinical judgment/standard of care.

**RESULTS:**

A total of 146 patients were included in the discussions of the MTB. The median age was 59 years, and 59.6% were women. Genomic results prompting a change in therapeutic decisions were obtained in 53.1% of patients (95% CI, 44.9 to 61.3). The most prevalent malignancy was non–small-cell lung cancer (51%). Other malignancies represented 60%, 50%, and 30% of patients with soft-tissue sarcomas, brain tumors, and breast cancer, respectively.

**CONCLUSION:**

Using an open-access virtual platform, MTBs were feasible in low- and middle-income countries on the basis of the capability to provide the benefits and access to available targeted therapies that are not standard of care. Furthermore, MTB recommendations were made available to the treating oncologist in different locations across Colombia, providing the option to modify clinical management in most of these patients.

## INTRODUCTION

Cancer diagnosis and treatment have dynamically evolved into a complex and multidisciplinary approach using next-generation sequencing (NGS). This technology is considered the leading diagnostic tool to find novel and well-known targets for genomic-based therapies, particularly in patients with limited response to the standard of care (SOC), especially in low- and middle-income countries (LMICs) where there are barriers to access to what is the SOC in developed countries.^1^ After clinical case evaluation, recommendations on the basis of molecular target actionability, tumor type, and patient characteristics are made available to the treating oncologist. This process facilitates an increasingly complex assessment of medical-scientific evidence about new biomarkers, and novel investigational medicines are incorporated into routine clinical practice, requiring interdisciplinary discussion to achieve treatment consensus.^2^

**CONTEXT**

**Key Objective**
Feasibility assessment: Can a virtual molecular tumor board (MTB) be implemented to improve access to precision oncology practice for patients with cancer in low- and middle-income countries?
**Knowledge Generated**
Multidisciplinary discussions via virtual MTB provided benefits and access to available targeted therapies that have the potential to change clinical management by making recommendations to treating oncologists on the basis of genomic profiling.
**Relevance**
Using an open-access virtual platform, multidisciplinary MTBs improved access to targeted therapies in low- and middle-income countries by connecting oncologists from remote and urban locations. In addition, clinical management recommendations were made available in a scalable and efficient manner to the treating oncologist to optimize outcomes for Hispanic patients with actionable genomic alterations.

Several commercial and noncommercial services have been developed, including a comprehensive genomic analysis of substitutions, insertion and deletion alterations (indels), and copy number variants in selected genes and gene rearrangements, as well as genomic signatures, including microsatellite instability and tumor mutational burden (TMB). Testing results interpretation using a multidisciplinary molecular tumor board (MTB) allows molecular alterations to represent tangible benefits for patients. As a result, they can be directed into enrollment in a clinical trial, expanded access programs, off-label indications,^3^ or other clinical management needs, such as further genetic consultation or testing on the basis of an inconclusive interpretation of the results.^4^ Consequently, implementing a MTB strategy improves access to targeted therapies, thus reducing the time to start treatment and less toxicity with improved patients' quality of life.^4-7^

Despite the exponential growth of scientific and technological development in precision oncology, LMICs still do not have equitable access to advanced life-saving technologies and cancer drugs deemed essential by oncologists.^8,9^ Moreover, barriers to delivering the most up-to-date treatment opportunities for patients with cancer in the context of a public health system further suggest the need for MTB not only to assess when comprehensive molecular testing would be indicated but also to evaluate novel therapeutic options for patients with newly discovered pharmacological alterations.^10^ Accordingly, some MTBs have been implemented in Latin America mainly for patients not responding to SOC systemic therapies.^11,12^ Herein, an open-access virtual MTB was implemented using the national cancer network to facilitate the evaluation of cancer and therapeutic approaches for Colombian patients.

## METHODS

### Study Details and Data Collection

**FIG 1:**
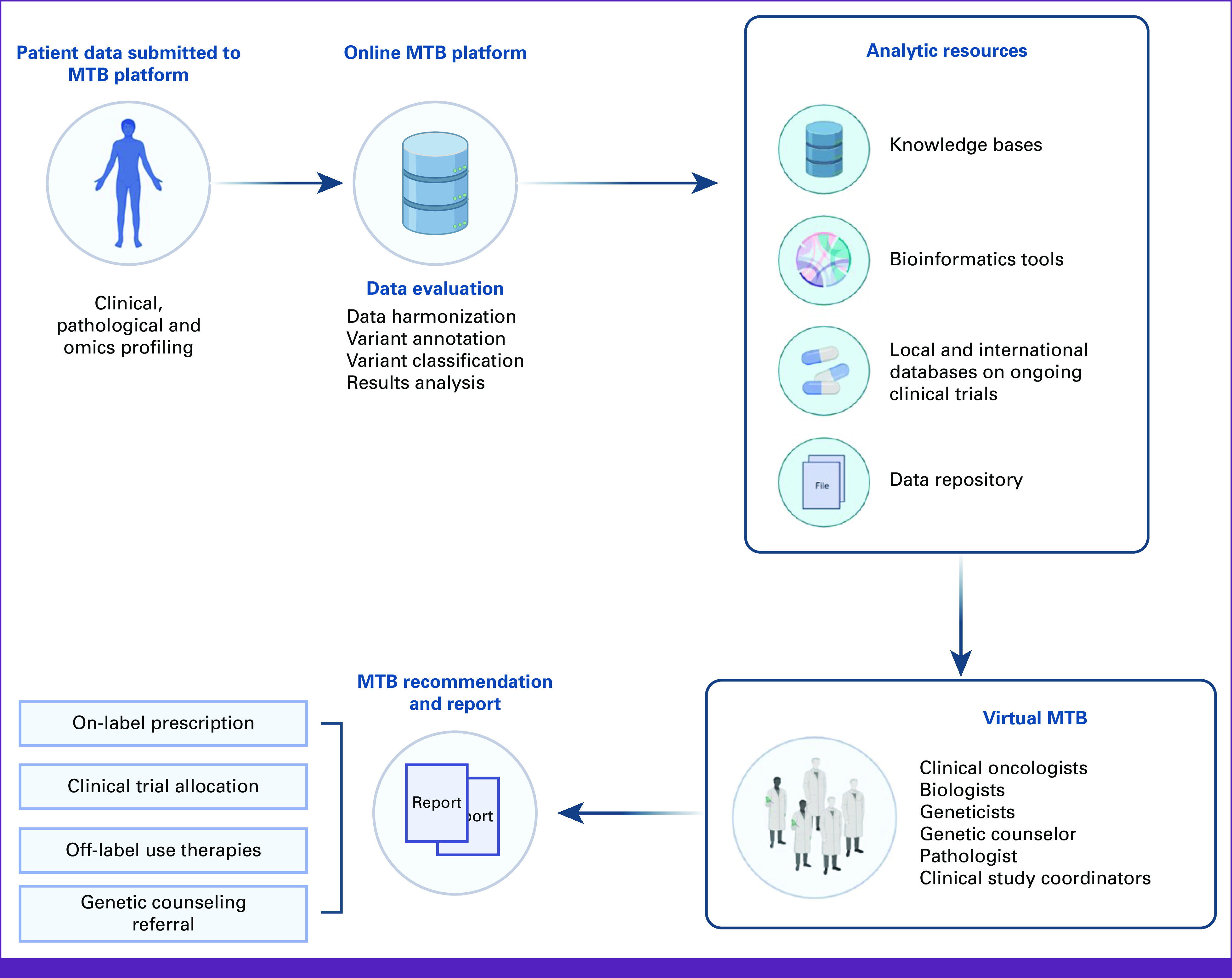
Genomic tumor board workflow. MTB, molecular tumor board.

A multidisciplinary MTB was created by The Foundation for Clinical and Applied Cancer Research (FICMAC) in Bogotá, Colombia, using an open-access virtual platform. Data from the first cohort of patients using this service was collected between August 2020 and November 2021. MTB's data repository includes demographics, histopathological reports, electronic medical records, results of molecular tests, and the clinical course of the disease. The clinical oncologists performed data entry into the electronic data capturing form across distinct locations in Colombia, using an online open-access virtual platform to be presented to the medical-scientific Committee (Fig 1). All patients provided informed consent and authorization to participate in the virtual MTB by providing relevant information while protecting personal information and anonymizing clinical records to the meeting attendees. To be eligible, patients were age older than 18 years and had a formalin-fixed paraffin-embedded (FFPE) tumor tissue slide or whole-blood samples for liquid biopsy analysis. This study was approved by a local Research Ethics Committee (Cayre Acta 2020-002-01, Jan-24/20-V4).

### MTB Organization and Workflow

The multidisciplinary MTB consisted of specialized physicians in clinical oncology, molecular pathology, genetic counseling, and clinical genetics and a molecular biologist trained in bioinformatics, cancer genetics, molecular oncology, and targeted therapies. In addition, some occasional attendees were physician-scientists and data scientists with oncogenic driver and signaling pathways expertise.

Three or four medical cases were discussed in scheduled virtual sessions of 1 hour per week, and the number of cases was gathered by clinical practice or external clinical reports requesting MTB recommendations. At the beginning of the MTB session, the treating oncologist described each patient, including sociodemographic characteristics (age and sex), Eastern Cooperative Oncology Group performance status, presence of comorbidities, and course of the disease (time of diagnosis, each treatment previously received, dates, and treatment response). Posteriorly, the assigned medical geneticists described the functional implications of the genomic alterations, particularly relevant, actionable pathways. Finally, on the basis of the medical history and NGS test reports, a course of clinical management was recommended and made available to the treating oncologist the option for targeted therapy.

The different recommendations on the basis of genomic profiling were classified as follows: first, the *diagnostic evaluation* provided for tumors of unknown primary location or metastatic lesions of patients in which clinical characteristics presented divergence from genomic profiling or for which conventional pathology failed to reach a conclusive etiologic diagnosis and the second recommendation consisted of a *new therapeutic standing* in which a driver mutation indication was newly found to match an available targeted therapy. These consisted of medications approved by the US Food and Drug Administration and the Colombian regulatory agency–the National Institute of Drug and Food Surveillance, which recommends following the ASCO guidelines for the patient treatment approach. Additionally, a commentary regarding pending approval was issued on *off-label indications* where medications were approved for other pathologies that shared the same molecular alteration as the reviewed tumor. The third recommendation consisted of *impactful findings*, including identifiable drug resistance and modulating alterations that could positively or negatively affect prognosis and survival.

For instance, *TP53* mutations, clonal hematopoiesis of undetermined potential, and rare translocations or amplifications were considered part of this category. When no targeted therapies could be recommended, evidence of modulation and prognosis with SOC and corresponding findings was given. The fourth type of recommendation consisted of germline or possible germline alterations. This recommendation was considered when liquid biopsies were performed, and a variant allelic fraction and an identifiable tumor-predisposing syndrome suggested a germline component. Therefore, the patient was referred to genetic counseling. A candidate cancer-predisposing mutation was informed if germline evaluation point mutations were detected. The final recommendation consisted of medication access. When patients were recommended, nonavailable therapies, expanded access programs or clinical trials were discussed as a plausible option.

Recommendation reports were sent to the treating clinical oncologist by confidential email with an encrypted password. Subsequently, on the basis of clinical judgment, the treating medical oncologist presented these recommendations to the patient and their family. If the patient's treatment was changed on the basis of the MTB recommendations, the new therapy was started only after the patient was informed and consented. However, we did not assess the treatment oncologists' compliance with MTB recommendations because they were not intended to replace clinical judgment and SOC in the Colombian Health system.

### Molecular Profiling Assays

Different assays from different vendors were offered on the basis of the insurance policy coverage for reimbursement. Although testing results were considered comparable, the possibility of false-negative results would vary depending on the distinct range of tested genes and RNA analysis resolution, which was reviewed on a case-by-case basis. Identified mutations and treatment recommendations reported on the assay results were normalized (ie, transcripts and mutation nomenclature). Additionally, mutations were independently reanalyzed for clinical action and resistance consulting OncoKB,^13^ ClinVar,^14,15^ the Catalogue of Somatic Mutations in Cancer (COSMIC),^15^ The Cancer Genome Atlas (TCGA),^16^ and cBio Portal for Cancer Genomics^17^ genomic data repositories.

### Statistical Analysis

Patient characteristics and demographic variables were summarized according to descriptive statistics using central tendencies and dispersion measures. Inferential statistics were used to conduct hypothesis testing and determine variable association. Time-to-event analysis was performed using the Kaplan-Meier methodology using the log-rank test for set comparisons. All analyses and graphs were elaborated on R studio (Version 4.2.1, The R Foundation, Vienna, Austria).

## RESULTS

### Demographic Characterization

**FIG 2:**
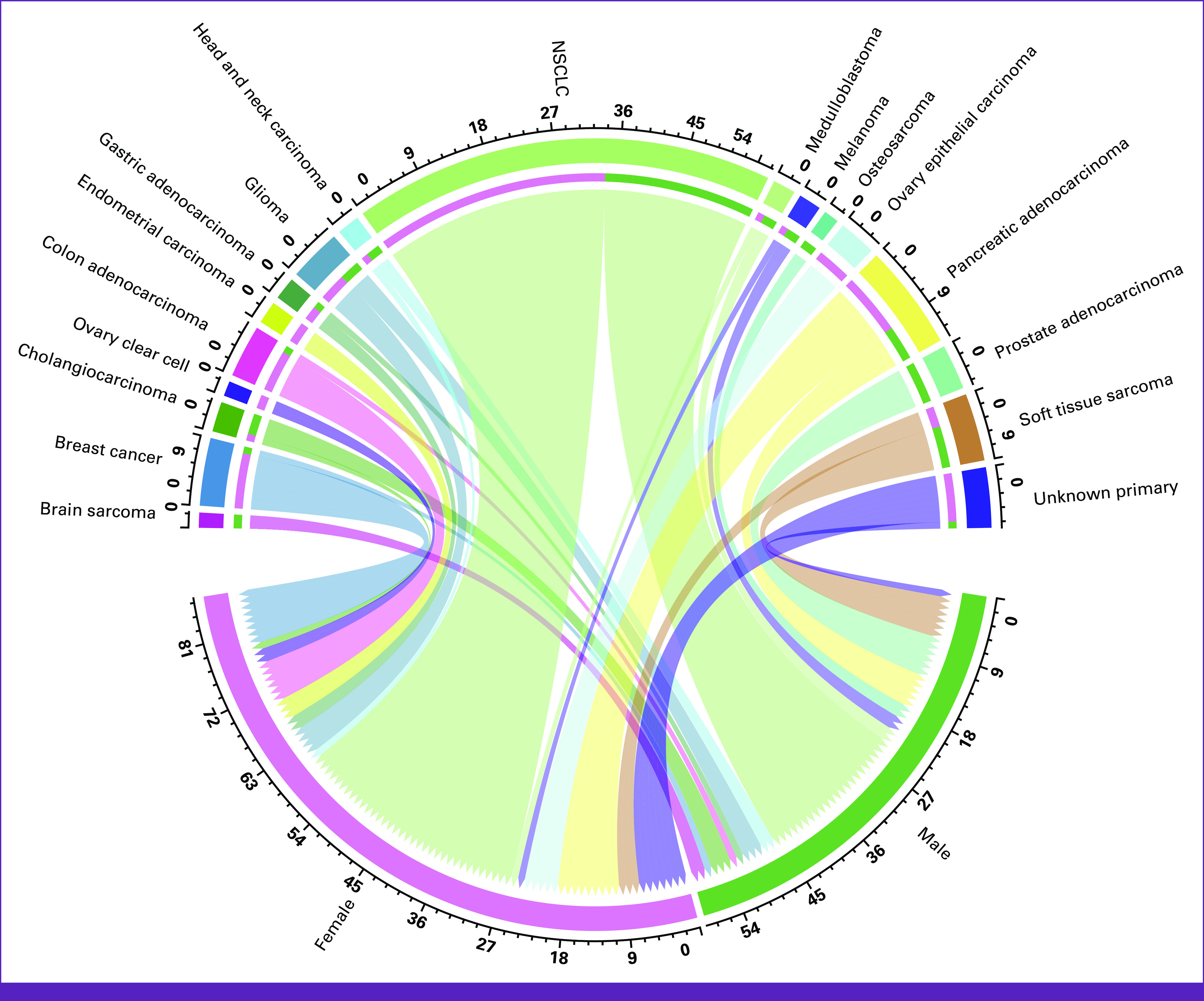
Cancer distribution and associated tumors by sex. Circular plot represents the tumors found by sex. Only frequent tumors (>two cases) are presented. NSCLC, non–small-cell lung cancer.

A total of 146 patients were included in the discussions of the MTB. The median age was 59 years, and the majority were women (59.6%). Additional clinical characteristics, including frequency of histological diagnoses and prior antitumor therapy, are included in Table 1. Pathologies considered rare were uveal and anal melanoma, pseudomyxoma peritonei, cervical adenocarcinoma, fallopian tube carcinoma, gallbladder adenocarcinoma, and a renal clear cell carcinoma, which were presented in one case each (Fig 2). These pathologies, when grouped, corresponded to seven cases of the cohort (4.8%).

**TABLE 1:** Characteristics of the Patients Analyzed in the Molecular Tumor Board and Included in the Study

Variable	Value (%)	95% CI or Range	
Female representation	59.6	51.6	67.5
Age, median, years	59	11	89
Tumor stage			
Complete remission	1 (0.68)	0	8.2
Early or localized disease	38 (26)	18.9	33.1
Metastatic disease	107 (73.3)	66.1	80.5
No. of previous lines			
0	29 (19.9)	13.4	26.3
1	40 (27.4)	20.2	34.6
2	41 (28.1)	20.8	35.4
3	14 (9.6)	4.8	14.4
>4	22 (15.1)	9.3	20.9
Reasons for referral			
Progressive disease after standard treatment	48 (32.9)	25.3	40.5
Rare tumor	58 (39.7)	31.8	47.6
Unknown	1 (0.68)	0	8.2
First-line definition	39 (26.7)	19.5	33.9
Primary lesion/histology			
Lung cancer	60 (41.1)	33.1	49.1
Adenocarcinoma	53 (88.3)	80.2	96.5
Sarcomatous	1 (1.6)	0	49
Adenosquamous	1 (1.6)	0	49
Enteric differentiation	3 (5)	0	10.5
Carcinoma of unknown primary	2 (3.3)	0	7.9
Pancreas	15 (10.3)	5.3	15.2
Ductal adenocarcinoma	13 (86.6)	69.5	100
Amphicrine carcinoma	1 (6.6)	0	19.3
Neuroendocrine	1 (6.6)	0	19.3
Brain	13 (8.9)	4.3	13.5
Medulloblastoma of the adult	3 (23.1)	0	45.9
Astrocytoma	5 (38.4)	12	64.9
Glioblastoma	2 (15.4)	0	34.9
Primary brain sarcoma	2 (15.4)	0	34.9
Esthesioneuroblastoma	1 (7.7)	0	22.2
Soft tissue	9 (6.2)	2.3	10.1
Undifferentiated pleomorphic sarcoma	5 (55.5)	23.1	88
Liposarcoma	2 (22.2)	0	49.4
Synovial sarcoma	1 (11.1)	0	31.6
Leiomyosarcoma	1 (11.1)	0	31.6
Breast ductal carcinoma	8 (5.5)	1.8	9.2
Ovary	8 (5.5)	1.8	9.2
Hig-grade serous carcinoma	6 (75)	45	100
Clear cell carcinoma	2 (25)	0	55
Prostate adenocarcinoma	6 (4.1)	0.9	7.3
Colon adenocarcinoma	5 (3.4)	0.5	6.4
Cholangiocarcinoma	5 (3.4)	0.5	6.4
Unknown primary	5 (3.4)	0.5	6.4
Gastric adenocarcinoma	4 (2.7)	0	5.4
Uterine	4 (2.7)	0	5.4
Endometrial carcinoma	3 (75)	32.6	100
Uterine leiomyosarcoma	1 (25)	0	67.4
Skin/melanoma	3 (2.1)	0	4.3
Head and neck	3 (2.1)	0	4.3
Bone	3 (2.1)	0	4.3
Osteosarcoma	2 (66.6)	13.3	100
Chordoma	1 (33.3)	0	86.7

**FIG A1:**
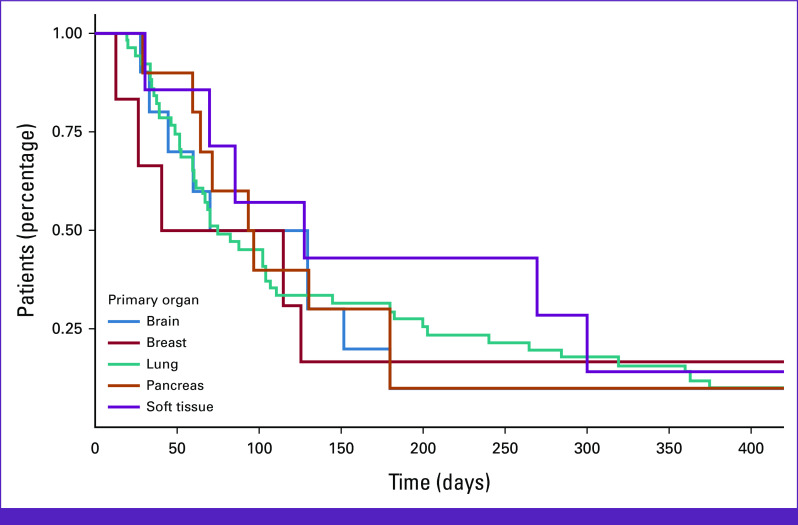
Time to referral based on primary tumor origin.

**FIG A2:**
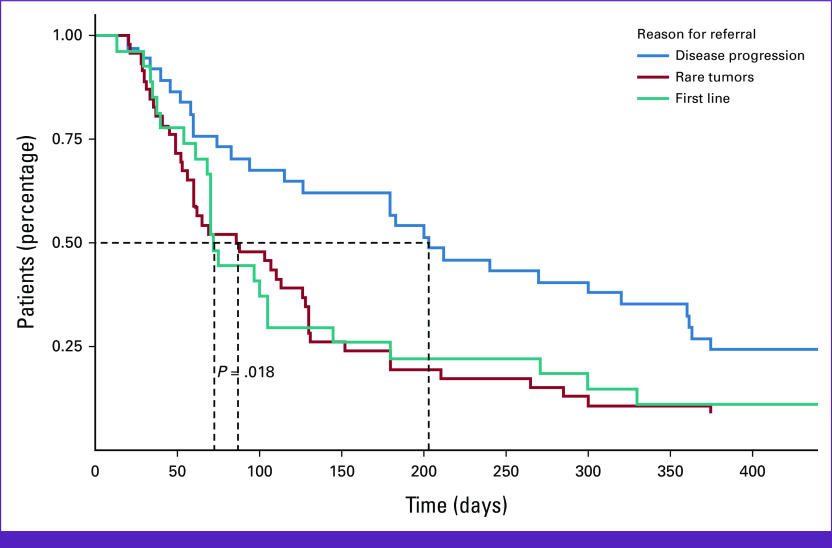
Time to referral based on reason.

Regarding referral times, the time from diagnosis to MTB meeting was a median of 104 days (95% CI, 75 to 130). No differences between tumor type and time to evaluations were found (*P* = .93) when considering the different pathologies and indications for genomic testing (Appendix Fig A1). As expected, patients who required first-line treatment allocation presented the shortest time to referral, comprising a median of 72 days (95% CI, 70 to 145), compared with 87 days (95% CI, 60 to 130) in the case of rare tumors and 203 days (95% CI, 127 to 361) in decision making after disease progression using SOC (*P* = .018; Appendix Fig A2). In addition, a correlation was identified between the number of previously received lines of treatment and time to MTB referral (*rho* = 0.37, *P* < .01).

### Genomic Results and TMB Analysis

**FIG 3:**
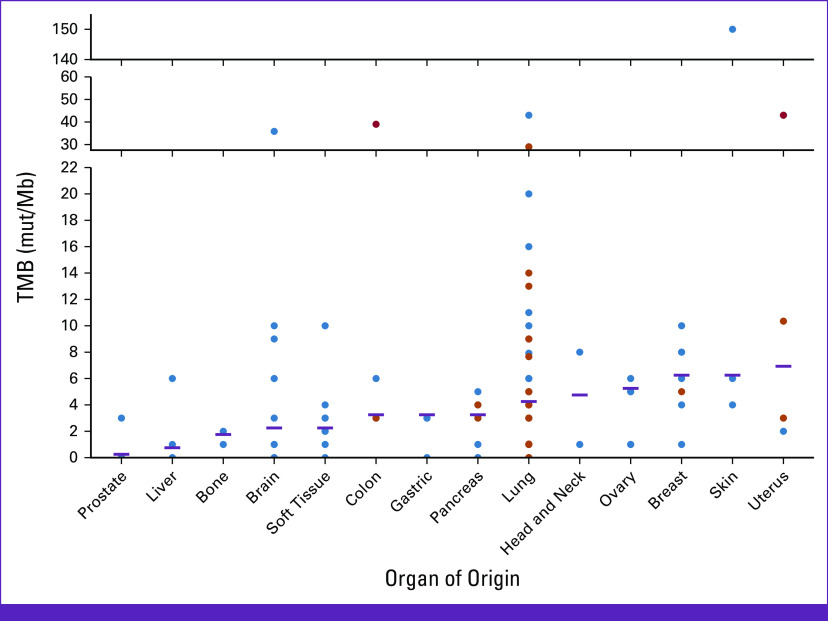
Representation of TMB across different organ origins. The image shows the patients (color points) and the median TMB (purple line) for each organ of origin (*x*-axis) and the regions where the TMB were measured (*y*-axis). mut, mutations; TMB, tumor mutational burden.

For genomic testing sample origin, a liquid biopsy was the only used methodology in 31 patients (21.2% [95% CI, 14.6 to 27.9]), tumoral tissue analysis in 105 (71.9% [95% CI, 64.6 to 79.2]), and both methodologies in 10 (6.8% [95% CI, 2.8 to 10.9]). Genomic results related to the number of actionable mutations were similar across pathologies (*P* = .265). The mean TMB across the cohort was 7.6 mutation per Mb, ranging from 0 to 150 mut/Mb, with the majority found in organs such as the lung, head and neck, ovary, breast, skin, and uterus (Fig 3).

### Treatment Decisions on the Basis of Genomic Test

**FIG 4:**
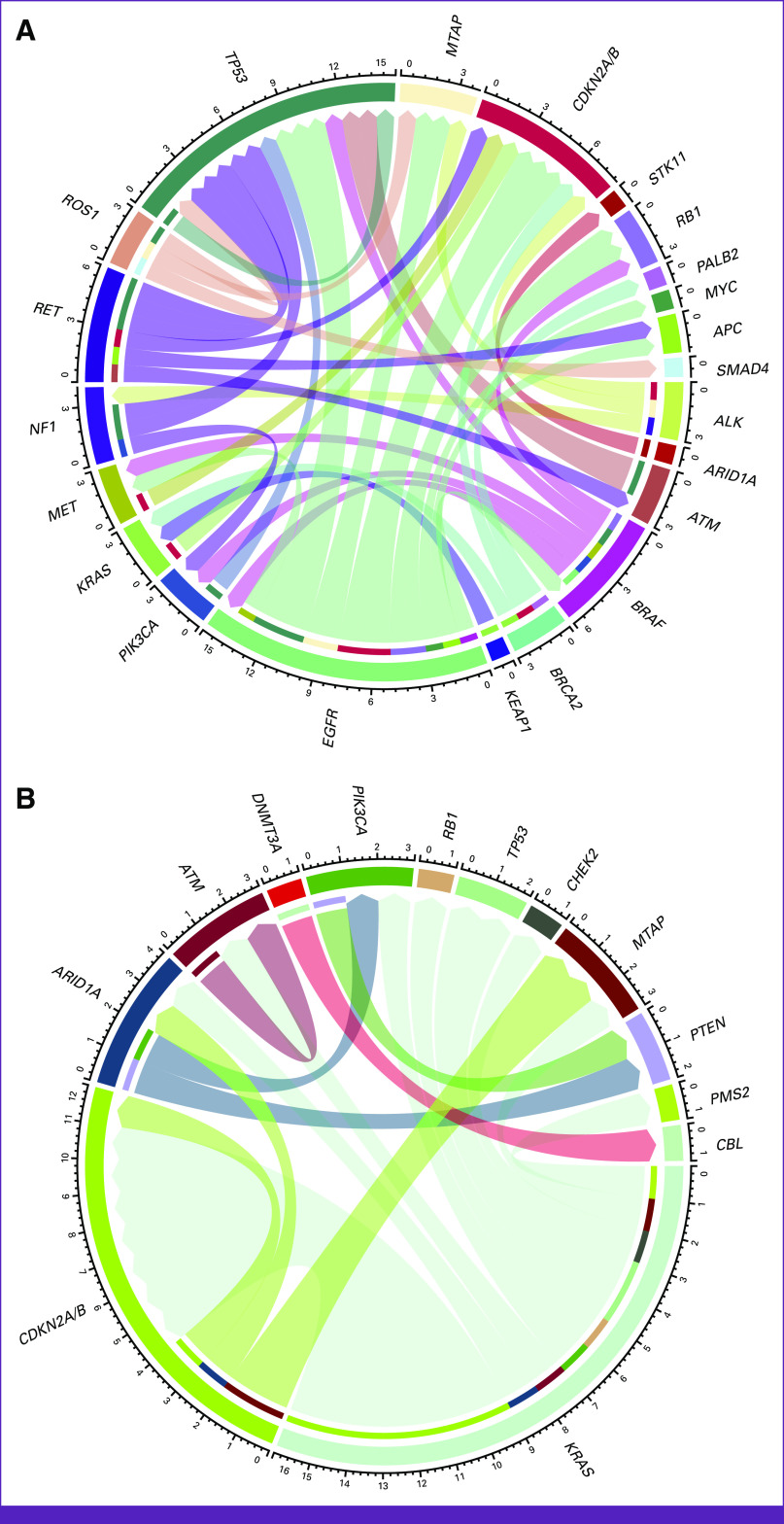
Co-occurring mutations among patients with (A) lung adenocarcinoma and (B) pancreatic ductal adenocarcinoma. Chord diagram arch corresponds to the co-occurrence. Considered driver mutations are depicted as the starting node.

**FIG A3:**
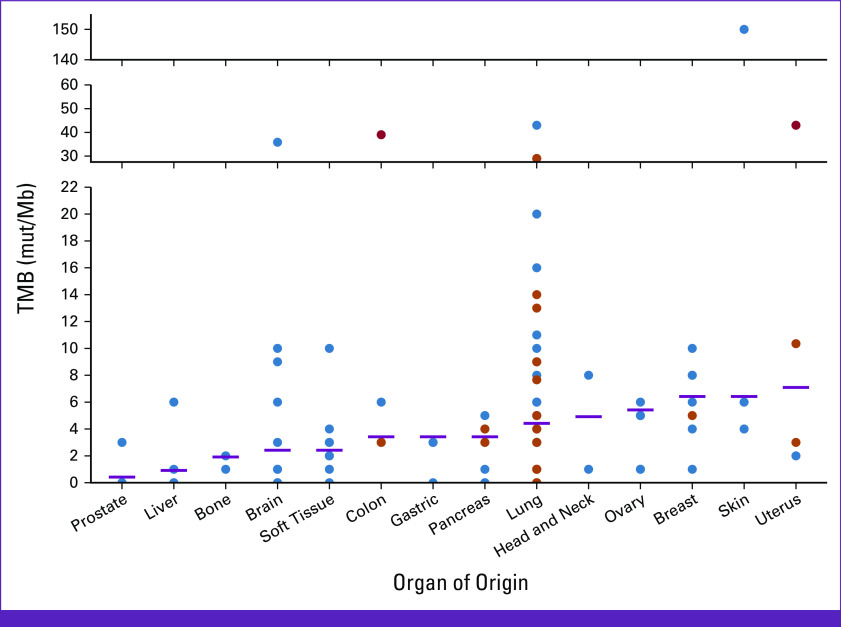
TMB by tumor type across patient samples. mut, mutations; TMB, tumor mutational burden.

Drivers and co-occurrent mutations of the two most frequent histology were analyzed (Figs 4A and 4B). Somatic analysis and clinical history revealed a possible germline cancer predisposing component in 32 patients (21.9% [95% CI, 15.2 to 28.6]), all referred to clinical genetics evaluation and genetic counseling. Seven patients had paired liquid biopsy evaluation, confirming a germline variant in the same study. In addition, pathogenic germline variants were found in *CDH1*, *ATM*, *DICER1*, *PMS2*, *TP53*, *STK11*, and *BRCA2*. Additional referred patients were subjected to complementary germline testing, in which a pathogenic variant in *APC* and a variant of unknown significance in *FANCM* were found (Appendix Fig A3).

Genomic results influencing treatment were obtained in 53.1% of patients (95% CI, 44.9 to 61.3), of whom 51% were patients with lung cancer. Considering individual pathologies, 67.2% of patients with lung cancer, 60% of patients with soft-tissue sarcomas, 50% of patients with brain neoplasms, and 30% of patients with breast cancer had treatment considerations modified on the basis of genomic testing. No differences favoring specific histology were found (*P* = .24). Furthermore, an alternative to the treatment recommendation given by the genomic testing report was proposed in 56.1% of cases (95% CI, 47.3 to 64.9). Interestingly, patients who experienced a favorable outcome were more likely to be given an alternative recommendation (71.2% *v* 39.1%, *P* < .001). Among patients with discordant recommendations, a significant increase in favorable outcomes was not observed in any specific pathology (*P* = .33).

## DISCUSSION

The multidisciplinary approach to cancer treatment solves the increasingly complex clinical scenario of obtaining extensive tumor genomic information derived from sequencing technologies to expand the SOC, which has recently been considered part of the oncology clinical practice in LMICs.^18^ NGS-based technology has been gradually introduced in the oncology practice in Colombia for the past 2 years. Herein, to our knowledge, the first MTB in Colombia to improve patients' access to targeted therapy and provide alternative options to optimize their clinical management is described.

Our study emphasizes the feasibility of MTB implementation in the therapeutic approach for oncology patients in Latin America. Of the 146 patients analyzed, the genomic profile obtained influenced treatment in more than 50%, most of whom were diagnosed with lung cancer. As non–small-cell lung cancer is one of the leading cancer diagnoses worldwide, it is important to consider molecular and multidisciplinary analysis before beginning treatment. Furthermore, targeted therapy is only available if in demand by a specific and well-supported request from the treating oncologist to receive reimbursement approval by the patient's insurance policy in Colombian medical care. At this point, 56% of patients had benefited from selecting the treatment guided by the comprehensive genomic evaluation. This last result agrees with the findings made by Huang et al^19^ which revealed that the benefit for patients was equal and independent of the health care scenario where the patients were treated: urban versus rural. According to that, MTB could be an effective strategy for improving access to health care among underrepresented populations, even in LMIC.

The first MTB was established in Latin America at the Alexander Fleming Institute in Argentina in 2019. Of the 32 patients analyzed by such MTB, 28 (87.5%) had potentially actionable alterations, and only four (12.5%) had no actionable mutation. Six (19%) patients received a drug recommendation approved by the local regulatory agency, and nine (28%) patients received an off-label authorized treatment recommendation.^12^ Other multidisciplinary panels have focused on recommendations for a specific type of cancer, such as breast cancer.^11^ Considering the size of the cohort evaluated in this MTB study and the frequency with which clinical management was changed on the basis of genomic function tests in different types of cancer, it can be considered that the scope of this study had a larger impact on the oncology's clinical practice in Colombia, supporting the notion that it is a pioneer study in the practice of precision oncology in Latin America. On a global scale, the real value of this MTB should be weighted at the patient level and beyond a single institution but considering the potential of MTB implementation as part of precision oncology clinical practice^7^—compared with a global scale.^20^

Implementing TMB is a valuable resource for clinical oncologists and patients with cancer in developing countries. Among its advantages is a better interpretation of tumor genetic profiles, drug resistance patterns, novel therapeutic options, or further genetic testing. However, in some LMICs, genomic profiling could be challenging, and techniques such as NGS face obstacles because of limited access.^20^ At the same time, the low penetrance of NGS makes the analysis of its reports even more difficult. The participation of multiple medical specialists at the MTB ensures expert opinion and a deep interpretation of the genomic profiling. The literature suggests that only a small fraction of patients, about 10% of sequenced patients, can reach clinical benefits from precision oncology.^21^ Nevertheless, this small fraction could be increased as MTB offers new treatment possibilities for the patients, including enrollment in novel clinical trials or consideration of alternative therapies to the SOC.^2^ The recommendations derived from an MTB include novel therapeutic approaches, such as off-label therapy and expanded access programs.^22^

The ongoing COVID-19 pandemic presented a novel challenge for the health care system worldwide, and almost all in-person consultations had to migrate to a virtual setting scenario. MTB meetings were performed on a virtual platform, a useful tool to connect many specialists simultaneously from different locations across Colombia. A German study showed that the virtualization of MTB during the COVID-19 pandemic facilitated various disciplines' participation and favored internal and external experts' presence in clinical case discussions.^23^ Some of the most important clinical gaps for personalized medicine implementation, according to Sadik et al,^24^ include treatment initiation without consideration of testing results or targeted treatment nonselection besides positive results. It could be possible that a more efficient MTB facilitated by information technology could close the gap and put oncology patients closer to their appropriate treatment. This study was proposed with open access for treating oncologists from different locations of the country to overcome the barrier of geographical accessibility. Additionally, it represented a unique educational opportunity for the medical professionals and the decision-making process of the treating oncologist on the basis of the final recommendations with the relative levels of evidence and the relevant bibliography used for the MTB. This could be one of the first steps to promote the implementation of MTB to reduce knowledge barriers in interpreting genomic tests and improve current clinical practice.

In conclusion, the results of this study demonstrate the feasibility of conducting a centralized open and widely available MTB and propose that patients with cancer would likely benefit from analyzing molecular profiles to identify potential targets for genomic-based therapies and other molecularly derived interventions.
